# Validation of TOF-SIMS and FE-SEM/EDS Techniques Combined with Sorption and Desorption Experiments to Check Competitive and Individual Pb^2+^ and Cd^2+^ Association with Components of B Soil Horizons

**DOI:** 10.1371/journal.pone.0123977

**Published:** 2015-04-20

**Authors:** Beatriz Cerqueira, Daniel Arenas-Lago, María Luisa Andrade, Flora A. Vega

**Affiliations:** Department of Plant Biology and Soil Science, Faculty of Biology, University of Vigo, Lagoas, Marcosende, Vigo, Pontevedra, Spain; NERC Centre for Ecology & Hydrology, UNITED KINGDOM

## Abstract

Sorption and desorption experiments were performed by the batch method on the B horizons of five natural soils: Umbric Cambisol, Endoleptic Luvisol, Mollic Umbrisol, Dystric Umbrisol, and Dystric Fluvisol. Individual and competitive sorption and desorption capacity and hysteresis were determined. The results showed that Pb^2+^ was sorbed and retained in a greater quantity than Cd^2+^ and that the hysteresis of the first was greater than that of the second. The most influential characteristics of the sorption and retention of Pb^2+^ were pH, ECEC, Fe and Mn oxides and clay contents. For Cd^2+^ they were mainly pH and, to a lesser extent, Mn oxides and clay content. The combined use of TOF-SIMS, FE-SEM/EDS and sorption and desorption analyses was suitable for achieving a better understanding of the interaction between soil components and the two heavy metals. They show the preferential association of Pb^2+^ with vermiculite, chlorite, Fe and Mn oxides, and of Cd^2+^ with the same components, although to a much lesser extent and intensity. This was due to the latter’s higher mobility as it competed unfavourably with the Pb^2+^ sorption sites. TOF-SIMS and FE-SEM/EDS techniques confirmed the results of the sorption experiments, and also provided valuable information on whether the soil components (individually or in association) retain Cd^2+^ and / or Pb^2+^; this could help to propose effective measures for the remediation of contaminated soils.

## Introduction

Increased concentrations of heavy metals of an anthropic origin in soils cause serious environmental pollution problems. These metals are present mainly due to factors such as industrial activities, mining, or solid urban waste [1]. Although soils can act as sinks for heavy metals, contamination problems can arise once their sorption capacity has been exceeded [2]. The mobility and bioavailability of heavy metals and the possibility of being transferred to other compartments of the ecosystem mainly depends on the sorption and desorption capacity of the different components of the soil [3]. The term sorption includes processes of adsorption, surface precipitation, and fixation, while desorption implies the release of sorbed species into the environment surrounding the soil particles [4]. The distribution of the metals amongst the soil components depends on the intrinsic properties of the types of metal involved, the soil properties, and the amount of metal added to it [5].

Lead mainly reaches soils through the use of fertilisers and pesticides, emissions from the combustion of coal and petrol, the dumping of industrial waste water and mining activities [6,7]. Cadmium mainly reaches the environment through atmospheric deposits; the application of phosphate fertilisers and manure, biosolids and industrial and mining waste [8,9].

Both metals become immobilised in soils as a result of a series of processes such as adsorption, chemical sorption, ion exchange, or surface precipitation. The results of different authors have shown that pH is the most important parameter to determine the mobility of Cd^2+^ [10]. Furthermore, the organic carbon, clay, Fe, Al, and Mn oxide contents, and the cation exchange capacity also favour the sorption of Cd^2+^ [11]. Mn oxides and organic matter are the components that most highly influence Pb^2+^ sorption [12,13].

Several studies have been published on sorption and desorption experiments with heavy metals; on the whole, they have looked into the distribution of metals between the soil and a solution of heavy metals after a contact period during equilibrium is achieved. Different authors have used sorption and desorption isotherms as well as the distribution coefficient Kd to compare the sorption capacities of different soils in identical experimental conditions [5,14–17]. In order to obtain results that would make it possible to compare the sorption and retention capacities, using different concentrations of metals in different soils, Vega et al. [3] proposed using the Kr parameters (partition coefficient) and HI (hysteresis). In this work, using these parameters, the individual and competitive sorption capacity for Pb^2+^ and Cd^2+^ and hysteresis were evaluated and compared in five subsurface horizons from different soils with significantly different characteristics.

In general, the use of isotherms and distribution coefficients provides estimates about the distribution of the heavy metals in the soil and equilibrium solution. However, this analysis does not provide real images that confirm the sorption of metals on the surface of the soil particles.

Sipos et al. [18] combined sorption experiments with analytical electron microscopy analyses to study in greater detail the association between metals and soil components. In a preliminary study, Cerqueira et al. [19] carried out individual sorption and desorption experiments for Cu^2+^ and competitive sorption and desorption experiments for Cu^2+^/Pb^2+^ in the Bt horizon of an Endoleptic Luvisol; they also used the TOF-SIMS technique to discover the distribution of Cu^2+^ and Pb^2+^ and how these interact with the different components of this soil, obtaining highly satisfactory preliminary results. In turn, it has been verified that the combined use of TOF-SIMS and FE-SEM-EDS is effective when identifying the affinity of soil components for different heavy metals in mine soils [20].

In this study, a series of mono and bimetallic sorption and desorption experiments were carried out, also studying the preferential distribution of the contaminating metals (^114^Cd and ^208^Pb) amongst the components of the B horizons of different soils.

The general purpose was to verify if the combined use of TOF-SIMS and FE-SEM/EDS, together with experiments on the monometallic and competitive sorption of Pb^2+^ and Cd^2+^, is suitable for identifying the association between each or both of the metals with the mineral components and their associations.

To this end, the specific aims were i) to exhaustively characterise the five B horizons of different soils, ii) to evaluate and compare the individual and competitive sorption and desorption capacity and hysteresis for both metals, and iii) to detect Cd^2+^ and Pb^2+^ on the surface of the soils and determine the interactions with the components and their associations by using TOF-SIMS and FE-SEM-EDS. After determining which have a greater influence on the retention of these metals it would therefore be possible to propose measures to control their mobility and bioavailability.

## Material and Methods

### Soils and sampling

Five natural soils in Galicia (NW Spain), developed on different parent matter, were selected to carry out this work. The five soil horizons studied were the subsurface ones of an Umbric Cambisol (UC) (N: 43° 44´ 33.1´´, O: 7° 42´4.8´´), an Endoleptic Luvisol (EL) (N: 43°030 43´ 54.3´´, O: 7° 55´16.2´´), a Mollic Umbrisol (MU) (N: 43° 33´ 1.8´´, O: 7° 20´2.4´´), a Dystric Umbrisol (DU) (N: 42° 09´ 264´´, O: 8° 50´ 739´´), and a Dystric Fluvisol (DF) (N: 42° 54´ 690´´, O: 8° 04´ 946´´) [21] developed on quartzite, amphibolite, slate, schist, and amphibolite, respectively. Specific permission was not required at these sampling locations and they did not endanger any protected species.

The samples from each subsurface horizon, namely UC.Bw, EL.Bt, MU.Bw, DU.Bw, and DF.Bw, were analysed and characterised in order to assess the influence of their components and properties on monometal and competitive Pb^2+^ and Cd^2+^ sorption, desorption, and hysteresis. An Eijkelkamp sampler was used to collect six samples from the B horizons of each soil, which were then stored in polyethylene bags. The samples from each horizon were subsequently pooled, air dried, passed through a 2-mm sieve and homogenized in a Fritsch Laborette 27 rotary sample divider. Each pooled sample was then split into twelve sub-samples, three of which were used for the soil analyses, three for the sorption and desorption experiments, three for FE-SEM/EDS, and three for TOF-SIMS.

### Soil analyses

Soil pH was determined with a pH electrode in 2.5:1 water/soil according to Slattery et al. [22]. Particle size distribution was determined after oxidising the soil organic matter with hydrogen peroxide, separating the coarser fraction (under 50 μm) by sieving, and using the finer fraction to carry out the internationally endorsed procedure [23]. The samples of the fraction less than 2μm (clay fraction) were saturated in Mg and calcined at 550°C. The clay fraction mineralogical analysis was performed in a Philips type powder diffractometer fitted with a Philips PW1710 control unit, a vertical Philips PW1820/00 goniometer and a FR590 Enraf Nonius generator (QL, 1%). The instrument was equipped with a graphite diffracted beam monochromator and copper radiation source [λ (Kα1) = 15,406 Å], operating at 40 kV and 30mA. The X-Ray powder diffraction pattern (XRPD) was collected by measuring the scintillation response to Cu Kα radiation versus the 2θ value over a 2θ range of 2–65, with a step size of 0.02° and counting time of 4 s per step. The determination was done by the RIR procedure (Reference method Intensity/Radio) using the corundum as a reference material [24]. The identification and quantification of the crystalline phases were performed using the Match! programme 2003–2012 Crystal Impact [25].

Specific surface area was determined by drying the samples at 110°C for 48 h, degasifying under vacuum for 4 h, and using approximately 1 g of degasified sample to obtain N_2_ sorption—desorption isotherms at −196°C and subatmospheric pressures in a Quantachrome Autosorb-6B apparatus; specific surface area was calculated by fitting the three-parameter BET equation (Murray et al., 1990) to the relative pressure *(P/P*_0_ *)* region 0.05–0.30 of the sorption isotherms.

Total organic C was determined with a TOC analyser-V CSH/CSN Shimadzu apparatus which performs the analysis by applying the principle of catalytic combustion oxidation and detection by non-dispersive IR [26]. The effective cation exchange capacity (ECEC) and exchangeable cation content were determined with the method of Hendershot and Duquette [27]. Aluminium, Ca, K, Mg, and Na were extracted with 0.1 M BaCl_2_, and their concentrations were determined by inductively coupled plasma optical emission spectrometry (ICP-OES) in a Perkin Elmer Optima 4300 DV apparatus.

Iron and Mn oxides contents were determined using the dithionite-citrate method [28, 29], and Al oxides content by the ammonium oxalate—oxalic acid method [30]. The samples were shaken with a solution of sodium hydrosulphite (0.5 g per gramme of soil) and sodium citrate (0.27 M) for Fe and Mn extraction, and with ammonium oxalate (0.2M) and oxalic acid (0.2M) for Al extraction. Fe, Mn, and Al concentrations in their extracts were determined by ICP-OES as above.

### Sorption and desorption experiments

Data for isotherm construction were obtained in batch experiments following the method described in Vega et al. [31]. Non-competitive sorption was evaluated using single-metal sorption solutions of Cd or Pb nitrates at concentrations of 0.01, 0.05, 0.1, 0.5, 1, and 3 mmol L^-1^; and competitive sorption using multi-metal solutions (Cd^2+^+Pb^2+^) in which each metal had the same concentration (again 0.01, 0.05, 0.1, 0.5, 1, and 3 mmol L^-1^). Both single-and multi-metal solutions also contained 0.01 M NaNO_3_ as the background electrolyte. The heavy metals were used in the form of nitrates because of the high solubility of these salts, and the concentrations were chosen to range from normal values to values representative of severe pollution. Triplicate suspensions of 6-g soil samples in 100 mL of solution in polyethylene tubes were shaken in a rotary shaker for 24 h at 25°C and then centrifuged at 5000 rpm. The resulting pellet was set aside for use in the desorption stage of the experiment. The supernatant was filtered through Whatman 42 paper and the resulting filtrate was analysed by ICP-OES in a Perkin-Elmer Optima 4300 DV apparatus (USA). The quantity of each metal that had been sorbed was calculated from the difference between its concentrations in solution before the addition to the soil and after equilibration (shaking) with the soil.

Sorption isotherms for each metal were constructed by plotting the sorbed metal content of the soil horizon (μmol g^−1^ dry soil) against the metal concentration in solution at equilibrium (μmol L^-1^).

Following Vega et al. [31], desorption experiments were conducted using the pellets obtained in the sorption phase of the experiments. The pellets were dried at 45°C and weighed; each pellet was shaken for 24 h in a polyethylene tube with 100 mL of 0.01 M NaNO_3_ solution at 25°C, which was then centrifuged at 5000 rpm. The supernatant was filtered through Whatman 42 paper and the resulting filtrate was analysed by ICP-OES. The quantity of each metal retained on the soil sample was calculated from the quantity sorbed (determined in the sorption stage of the experiment) and the concentration of the metal in solution following desorption.

The desorption isotherms for each metal were constructed by plotting the amounts of metal retained in the soil horizons (μmol g^−1^ dry soil) against the metal concentration in solution following desorption (μmol L^−1^). The sorption and desorption isotherms were compared, whenever possible, to the types of curve described by Giles et al. [32].

For both sorption and retention data, and in both cases for the competitive and non-competitive situations, the parameter *K*_r_ [3] was calculated as follows. The sorption data were fitted with equations of the type:

$${\text{C}}_{s,i}={\text{K}}_{\text{r}1}{\text{C}}_{p,i}$$ 1

and

$${\text{C}}_{p,i}-{\text{C}}_{s,i}={\text{K}}_{\text{r}2}{\text{C}}_{p,i}$$ 2

where *C*_s,*i*_ is the amount of metal *i* that was sorbed per gram of soil and *C*_p,*i*_ is the amount of metal *i* that was potentially sorbable, i.e. the amount in the initial sorption solution (per gram of soil). *K*_r_ was then defined as *K*_r1_ if the coefficient of determination of the first equation was larger than that of the second and 1 − *K*_r2_ otherwise. When thus calculated from sorption data, *K*_r_ varies from 0 for totally non-sorbent soils to 1 for a soil that completely eliminates metal *i* from solution. The capacity for retention of sorbed metal in desorption experiments, *K*_r_ was calculated in the same way as for sorption, except that the sorbed metal was replaced by metal retained at equilibrium; in this case *K*_r_ is 0 for a soil that completely releases all sorbed metal, and for a soil that releases no metal during the desorption phase of the experiment, it adopts the value obtained using the corresponding sorption data.

Sorption irreversibility was measured by a hysteresis index (HI), defined as the ratio between the *K*r values for retention (*K*r,r) and sorption (*K*r,s) [33]:

$$\text{HI}=\frac{K\text{r},\text{r}}{K\text{r},\text{s}}$$ 3

HI is equal to 1 if sorption is completely irreversible, and 0 if all of the sorbed metal is released.

### Analysis of untreated soil samples and samples after desorption of Pb^2+^ and Cd^2+^ by TOF-SIMS

A TOF-SIMS IV instrument from Ion-TOF was used to investigate the elemental and molecular structure of the samples and to obtain a clearer understanding of the chemical composition and location of the species present on the surface of the samples.

The TOF analyzer separates the ions according to the time they take to travel along the length of the field-free flight-tube. This time interval is related to the mass and charge of the accelerated particles. The energy and angular dispersion of the secondary ions can be compensated using focusing elements such as a reflectron. The lighter secondary ions arrive before the heavier ones, whereby a mass spectrum can be recorded. TOF-SIMS works by focusing and scanning a narrow pulsed ion beam on the surface. This process leads to the emission of charged secondary ions in a sputtering process from the outermost surface of the sample. Further analysis of the secondary ions provides information on the molecular and elemental species and their isotopes present on the surface. The secondary ions collected and represented in the mass spectra can be attributed to complete molecules, large fragments of molecules that have only lost functional groups. In this study, to increase our knowledge of the sorption and retention of Pb^2+^ and Cd^2+^ in the soil, we used this technique.

TOF-SIMS analysis was performed with untreated B horizon samples and those samples obtained after desorption experiments. During the TOF-SIMS experiment, the sample was bombarded with a pulsed bismuth ion beam. The secondary ions generated were extracted at a voltage of 10 kV, and their time of flight from the sample to the detector was measured in a reflection mass spectrometer. The analysis conditions for this study were 25 keV pulsed Bi^3+^ beam at 45° incidence, rastered over 500×500 μm^2^ at a square pixel density of 256×256, and 50 accumulative scans in each analysed area. The operating pressure in the main chamber was 5 × 10^–10^ mbar. An Electron Flood Gun (low energy electrons) was used to compensate the surface charge build-up process during the experiment. Positive secondary ion mass spectra were acquired over a mass range from m/z = 0 to m/z = 1000. The mass resolution (m/Δm) of the secondary ion peaks in the positive spectra was typically between 3600 and 6000. Before further analysis, the positive spectra were calibrated using CH_3_^+^, C_2_H_3_^+^, C_3_H_5_^+^, and C_7_H_7_^+^ ions. To obtain two-dimensional imaging (chemical surface maps), polyatomic bismuth projectiles (Bi^3+^) were focused onto the surface in a rastered mode. The intensities detected for secondary ion signals were colour-coded according to a scale. The chemical maps produced by TOF-SIMS represent the ions that reached the detector, rather than the ions that were present on the surface. The intensities cannot be used to derive absolute surface concentrations, as each solid has its own ability to release ions. However, the chemical maps are very useful to indicate the relative surface abundance and how it changes depending on the time or sample treatment. The studied ions were Al, Si, Mn, Fe, Si, and C_3_H_5_^+^ (organic matter) as representative of the main soil components, and Pb^2+^ and Cd^2+^ were the added metal ions.

The intensity bar to the right of each image is indicative of the signal intensity of each ion. Black means no signal or, therefore, points of minimal or zero presence of the ion. White indicates maximum signal strength and, therefore, points of maximum ion abundance. The bottom left of each image shows the ion corresponding to the intensity distribution map and the TC value is below it. The number of total counts of each ion is represented by an MC value. The images are overlapped to determine the matching or non-matching rows in the ions distribution on the surface. When ions overlap this may or may not lead to areas in the last image in which only one colour can be seen. In the samples coloured in red, blue and green and in their overlaps, the different combinations of colours indicate the coincidences (or not) in the distribution of the ions on the different surfaces of the soil components. Overlapping the images makes it possible to see the areas where there is a secondary colour produced by the combination of red + green = yellow, red + blue = purple, and green + blue = cyan. The point where the ions coincide produces white areas.

### Soil analysis by scanning electron microscopy

Soil samples were examined using a JEOL JSM-6700 f plus FE-SEM with charge compensation for all applications in both conductive and non-conductive samples. The FE-SEM was equipped with an Energy Dispersive Spectrometer (EDS), and the mineral identification was made on the basis of morphology and grain composition, using both secondary electron and back-scattered electron modes. Samples were set on a standard aluminium slide with carbon adhesive, coating them with layers of carbon of 20-nm thick. EDS spectra were recorded in the FE-SEM image mode.

The soil samples were analysed after the desorption process due to the importance of knowing the distribution between soil particles, especially in the case of high sorption hysteresis such as that found in the soil studied for both metals.

### Statistical analyses

All analyses were performed in triplicate. The data obtained in the analytical determinations were analysed with the statistical program IBM-SPSS Statistics 19.The results shown for of the soil analyses are the average of three determinations and they are expressed on a dry material basis. The significance of differences between means was estimated by variance analysis (ANOVA), followed by least significant difference (LSD) tests. The influence of soil characteristics on sorption and retention capacities was investigated by means of the pairwise Pearson correlation, as well as correlation analysis between the horizon characteristics and hysteresis indices.

## Results and Discussion

### Soil characteristics

Statistical analysis showed that there were significant differences in the components and properties that most influence the sorption capacity, the mobility and therefore the retention of metals by the soils (Table 1). The pH of the soils varied between 4.7 (UC.Bw) and 6.4 (EL.Bt) and the total organic carbon content varied between 67.2 (DU.Bw) and 8.7 g kg^-1^ in EL.Bt. The horizon Bt from soil EL had the highest proportion of Fe and Mn oxides and the lowest of Al oxides (Table 1). The Mn oxides content was low, whereas the content of Fe oxides was generally the highest of the free oxides (Fe, Mn and Al) (Table 1). The effective cationic exchange capacity (ECEC) was very low in all of the horizons except in the EL.Bt horizon, due to the high exchangeable Mg^2+^ content. The sand fraction dominated in all the horizons in the EL.Bt horizon, whose clay content was very high. The mineralogical analysis of the soil fraction < 2 μm (namely the clay fraction) showed that, except in the EL.Bt, DF.Bw and DU.Bw horizons, with a predominance of vermiculite, gibbsite, and quartz respectively, the most abundant mineral was kaolinite (S1 Fig). These results showed that selected soils were suitable for comparing the competitive and individual sorption and desorption capacity of Pb^2+^ and Cd^2+^. The specific surface area, which had a great influence on the sorption and fixation of the studied metal ions, varied widely among the different soils (13 to 78 m^2^ g^-1^), and the highest corresponded to the soil with the greatest clay content (S2 Fig).

**Table 1:** Characteristics of soils.

Soil characteristics		Units	Horizon				
			UC.Bw	EL.Bt	MU.Bw	DU.Bw	DF.Bw
pH_H2O_			4.7d	6.4a	5.2c	5.0c	5.4bc
TOC		g kg^-1^	21.7c	8.7d	47.4b	67.2a	16.8c
FeOx			17.0d	30.1a	29.0b	23.8c	4.3e
MnOx			0.01c	0.36a	0.20a	0.05b	0.01c
AlOx			12.1b	4.4d	20.8a	6.8c	11.5b
ECEC		cmol_(+)_kg^-1^	3.1b	62.2a	3.0b	5.4b	1.8b
Exchangeable	Na^+^		0.16c	0.69a	0.34b	0.45ab	0.07c
	K^+^		0.24a	0.22a	0.15b	0.13b	0.03c
	Ca^2+^		0.20b	2.7a	0.13b	1.1b	0.05b
	Mg^2+^		0.11b	58.4a	0.21b	0.89b	0.02b
	Al^3+^		2.4a	0.20c	2.1ab	2.9a	1.6b
Specific surface area		m^2^ g^-1^	25b	78a	14c	24b	13c
Sand		%	45.6d	21.3e	60.9b	66.3a	60.3c
Silt			23.9a	12.6c	28.3a	17.6b	28.4a
Clay			30.4b	66.1a	10.8d	16.1c	11.4d
Clay mineral fraction: Semiquantitative mineralogical analysis	Vermiculite	%	-	XXXX	XX	X	-
	Chlorite		-	X			
	Mica		tr	-	X	XXX	-
	Kaolinite		XXX	X	XXX	X	XXX
	Quartz		XXX	tr	XXX	XXXX	tr
	Gibbsite		XXX	-	-	X	XXXX
	Plagioclase		-	-	X	-	-

TOC: total organic carbon, FeOx: iron oxides, MnOx: manganese oxides, AlOx: aluminium oxides, ECEC: effective cation exchange capacity. tr:< 3%, X: 3–10%, XX: 10–30%; XXX: 30–50%, XXXX: >50%. For each parameter, values followed by different letters differ significantly with P < 0.05.

### Individual and competitive sorption and desorption isotherms

**Fig 1:**
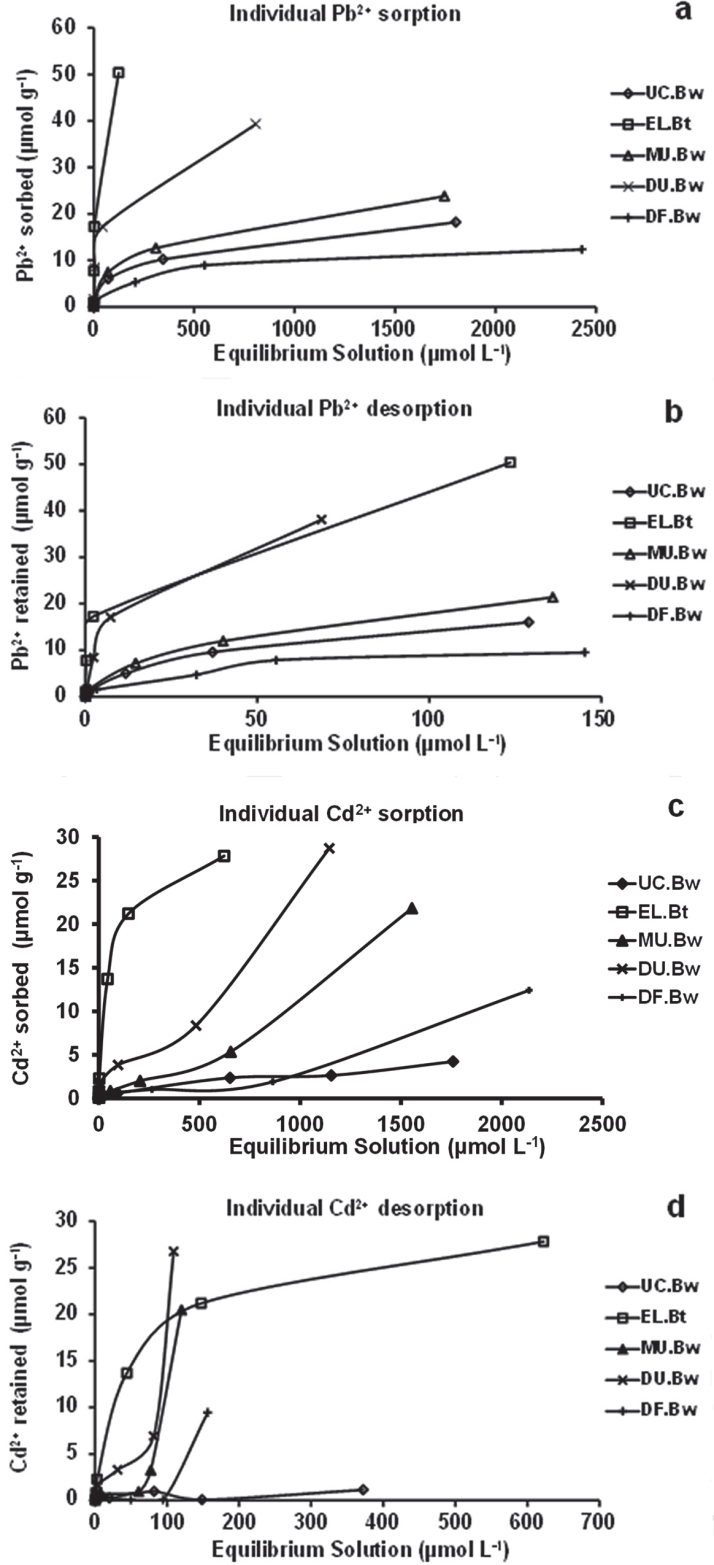
Individual sorption (a and c) and desorption (b and d) isotherms of Pb^2+^ and Cd^2+^.

**Fig 2:**
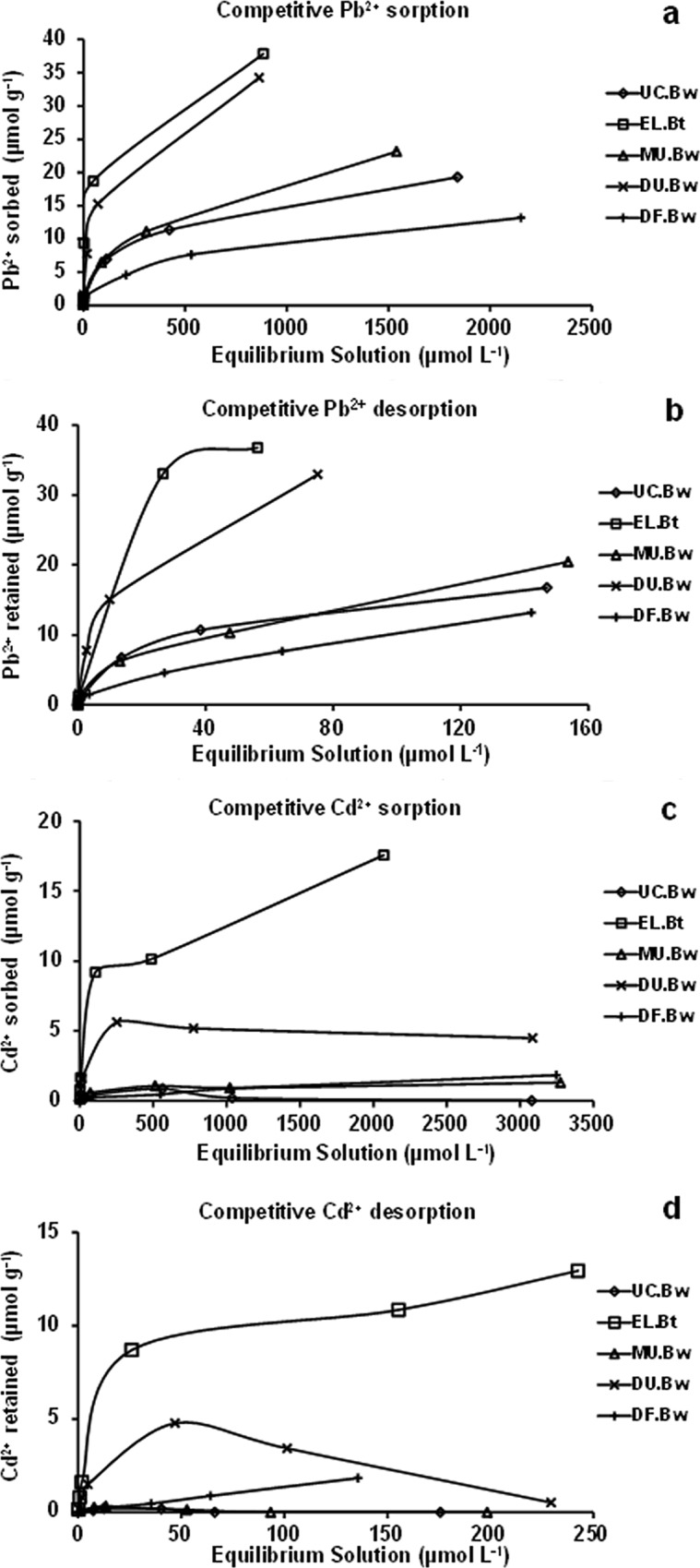
Competitive sorption (a and c) and desorption (b and d) isotherms of Pb^2+^ and Cd^2+^.

The individual and competitive sorption and desorption isotherms are shown in Figs 1 and 2. The individual and competitive sorption isotherms of Pb^2+^ for the horizons of MU.Bw, UC.Bw, and DF.Bw were type L (Figs 1A and 2A), indicating that Pb^2+^ had a relatively high affinity for these horizons. On the other hand, the individual and competitive sorption isotherms of Pb^2+^ for DU.Bw and EL.Bt were type H (Figs 1A and 2A), or high affinity. They indicate that Pb^2+^ had an extraordinarily high affinity for these soils, leading to the initial slope being very high. Individual desorption isotherms of Pb^2+^ were similar to those of sorption for each of the horizons studied. In horizons MU.Bw, UC.Bw, and DF.Bw, they were L type, and those of EL.Bt and DU.Bw were H type (Fig 1B). After desorption, the Pb^2+^ concentration in the equilibrium solution was low, mainly in DU.Bw and EL.Bt, (isotherms H type), showing that Pb^2+^ was strongly retained in the soils (Fig 1B). Moreover, competitive desorption isotherms of Pb^2+^ were L type for the all horizons (Fig 2B), showing that Pb^2+^ was also retained in the soils. In general, the majority of the individual and competitive sorption and desorption isotherms for Cd^2+^ of the horizons DU.Bw, MU.Bw, UC.Bw, and DF.Bw were not comparable with the types of curves described by Giles et al. [32] (Figs 1C and 1D, 2C and 2D). In turn, the sorption and desorption isotherms of Cd^2+^ were H for EL.Bt, showing that this horizon had higher affinity than the aforementioned for this heavy metal (Figs 1C and 1D, 2C and 2D).

### Sorption and desorption capacities and hysteresis

As a result of these irregularities, some isotherms did not fit the different empirical models, and the calculations for the sorption and retention capacity using the distribution coefficients *K*_*d*_ (L kg^-1^), as proposed by different authors [14,34], did not make it possible to clearly discriminate and compare the individual and competitive sorption capacities.

Therefore, the best parameter to estimate the sorption and retention capacity is the parameter *K*_*r*_ proposed and validated by Vega et al. [3]. Table 2 shows the Kr values for the individual and competitive sorption and retention capacities for Pb^2+^ and Cd^2+^.

**Table 2:** Kr for the individual and competitive sorption and retention of Pb^2+^ and Cd^2+^ and hysteresis indices.

	Kr sorption			
Soil	Pb^2+^ individual	Pb^2+^ competitive	Cd^2+^ individual	Cd^2+^ competitive
UC.Bw	0.406d	0.419c	0.182d	0.005d
EL.Bt	0.962a	0.739a	0.775a	0.359a
MU.Bw	0.476c	0.495b	0.440bc	0.028c
DU.Bw	0.769b	0.730a	0.589b	0.112b
DF.Bw	0.255e	0.280d	0.233c	0.032c
	Kr retention			
Soil	Pb^2+^ individual	Pb^2+^ competitive	Cd^2+^ individual	Cd^2+^ competitive
UC.Bw	0.362d	0.371c	0.028d	0.001b
EL.Bt	0.956a	0.722a	0.700a	0.278a
MU.Bw	0.432c	0.442b	0.399bc	0.001b
DU.Bw	0.749b	0.705a	0.543b	0.039b
DF.Bw	0.204e	0.225d	0.167c	0.001b
	Hysteresis indices			
Soil	Pb^2+^ individual	Pb^2+^ competitive	Cd^2+^ individual	Cd^2+^ competitive
UC.Bw	0.892d	0.886b	0.156c	0.152b
EL.Bt	0.994a	0.977a	0.903a	0.774a
MU.Bw	0.908c	0.892b	0.909a	0.022c
DU.Bw	0.973b	0.967a	0.923a	0.352ab
DF.Bw	0.799e	0.803c	0.718b	0.001d

For each parameter in each column values followed by different letters differ significantly with P < 0.05.

The results showed that the horizon EL.Bt (whose Kr was closest to 1) had one of the greatest capacities for individual and competitive sorption of Pb^2+^ and Cd^2+^. It also had the highest retention capacity of both metals after the desorption step (Table 2). This is attributable to the fact that this was the horizon with highest pH, ECEC and iron and manganese oxides, clay content, and specific surface area (Table 1). Their influence on the fixation of both ions was also confirmed after the correlation analysis. The sorption and retention capacity was correlated with the above mentioned parameters (Table 3).

**Table 3:** Correlations between soil characteristics and the Kr for individual and competitive sorption or retention of Pb^2+^ and Cd^2+^.

	Pb^2+^ individual		Cd^2+^ individual		Pb^2+^ competitive		Cd^2+^ competitive	
	Kr,s	Kr,r	Kr,s	Kr,s	Kr,s	Kr,r	Kr,s	Kr,s
pH_H2O_	0.605*	0.608*	nc	0.685**	Nc	Nc	0.882**	0.782**
FeOx	0.765**	0.759**	0.533*	0.730**	0.802**	0.788**	0.532*	Nc
MnOx	0.509*	0.510*	Nc	0.667**	Nc	Nc	0.781**	0.701**
AlOx	Nc	Nc	Nc	Nc	Nc	Nc	Nc	Nc
ECEC	0.744**	0.777**	Nc	0.688**	0.599*	0.612*	0.954**	0.775**
Clay	0.714**	0.717**	0.708**	Nc	0.541*	0.557*	0.872**	0.817**
Ssa	0.818**	0.821**	0.620*	0.660**	0.657**	0.672**	0.956**	0.872**

Kr,s: Kr sorption; Kr,r: Kr retention. Ssa: Specific surface area. Nc: No correlation.

**The correlation is significant at the level of 0.01 (bilateral).

*The correlation is significant at the level of 0.05 (bilateral).

These results were consistent with previous studies [13,31] where showed that Fe and Mn oxides have a high affinity for Pb^2+^. In addition, the high clay content of this horizon, particularly chlorite [35] and vermiculite [31], and the pH near neutrality also contributed to Pb^2+^ fixation. The statistical analysis confirmed the existence of significant correlation between clay content, pH and Pb^2+^ sorption capacity of Pb^2+^ (Table 3).

The low Pb^2+^sorption and retention capacities of the UC.Bw horizon were due to its acidity, low Fe and Mn oxide content, and the mineral composition of its clay fraction, with a predominance of kaolinite and quartz, minerals with a low sorption capacity. On the other hand, the low capacities of the DF.Bw horizon were due to it had the lowest Fe and Mn oxide contents and the lowest ECEC.

The individual sorption capacity for Pb^2+^ of MU.Bw was slightly higher than that of UC.Bw and much higher than that of DF.Bw. These differences were even greater in the case of the individual sorption of Cd^2+^ (Table 2). Similar results were obtained for the competitive sorption of Pb^2+^ compared to Cd^2+^, in which the sorption capacity of Pb^2+^ in MU.Bw was higher than in UC.Bw and DF.Bw. The Kr corresponding to the competitive sorption of Cd^2+^ compared to Pb^2+^ was very low, indicating that when competes with Pb^2+^, Cd^2+^ was scarcely sorbed and it remained in the equilibrium solution.

UC.Bw, MU.Bw and DF.Bw had a slightly higher competitive sorption capacity for Pb^2+^ than an individual capacity, showing a synergy of Pb^2+^ in the presence of Cd^2+^ [36] (Table 2). In general, the small amount of Cd^2+^ that was sorbed and retained in the presence of Pb^2+^ confirmed that Pb^2+^ competed favourably for the sorption sites with Cd^2+^.

A number of authors have referred to the high mobility of Cd^2+^ in soils, especially those with a lower pH. Zheng et al. [37] found that the sorption of Cd^2+^ is affected by pH and the presence of other cations, and Alloway [38] and Adriano [8] also showed that the mobility of Cd^2+^ is preferably controlled by the pH. This is also confirmed with the highly significant correlation established between pH and Cd^2 +^ fixation (Table 3).

The selectivity sequences for the competitive sorption and desorption show that in all of the soils, the sorption and retention of Pb^2+^ were higher than for Cd^2+^, therefore Pb^2+^ preferably occupied the sorption sites.

Table 2 shows the hysteresis indices and the irreversible fixing sequences of Pb^2+^ and Cd^2+^, obtained by means of ANOVA and LSD analyses. The results indicate that i) the horizons with the highest hysteresis indices were EL.Bt and DU.Bw, both for the individual and for the competitive treatment with Pb^2+^, and that the irreversibility was slightly lower in the second case, except for DF.Bw; ii) the Cd^2+^ in competition with Pb^2+^ had the lowest HI, indicating the higher mobility of this cation when it competed with Pb^2+^; (iii) the hysteresis of the individual sorption of Cd^2+^ was very high in comparison to the competitive sorption, with horizons EL.Bt, DU.Bw and MU.Bw having the highest irreversibility indices; (iv) the most reversible sorption for each treatment and metal (except for individual Cd^2+^) occurred in horizon DF.Bw, which had the lowest ECEC, specific surface area, and Fe and Mn oxides content.

The highest hysteresis occurred in horizon EL.Bt, which, as mentioned above, had the highest pH, a high content of clays, Fe and Mn oxides, a high ECEC and specific surface area. The second highest hysteresis was in DU.Bw, also with a high Fe oxide content and the highest organic carbon content (Table 1). UC.Bw had the lowest pH, DF.Bw the lowest Fe and Mn oxides content and both had the lowest hysteresis (Tables 1 and 2).

### TOF-SIMS experiments

Analyses by TOF-SIMS were carried out before the treatments and after desorption stage in order to verify the spatial distribution of the retained Pb^2+^ and Cd^2+^ and their association with the different soil components.

**Fig 3:**
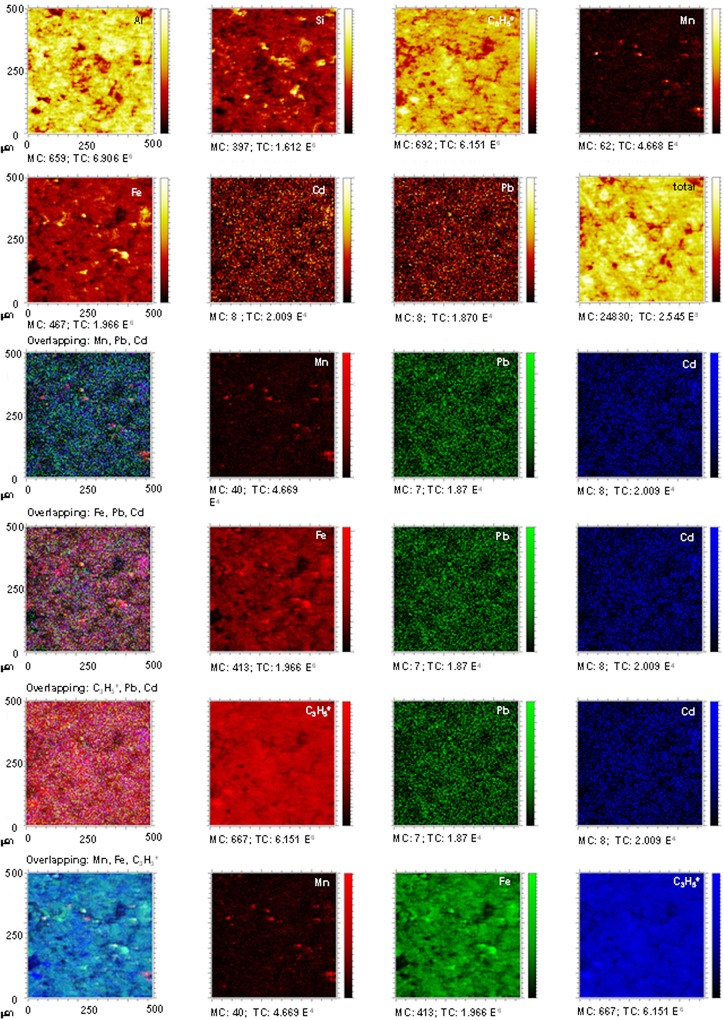
Untreated DU.Bw horizon. TOF-SIMS images of Al, C_3_H_5_^+^,Si, Fe, and Mn ions, and overlapping all the signals with Pb^2+^ and Cd^2+^. Images of overlapping: Mn + Pb + Cd; Fe + Pb + Cd; C_3_H_5_^+^ + Pb + Cd; and Mn + Fe + C_3_H_5_^+^ showing the concordance between signals.

**Fig 4:**
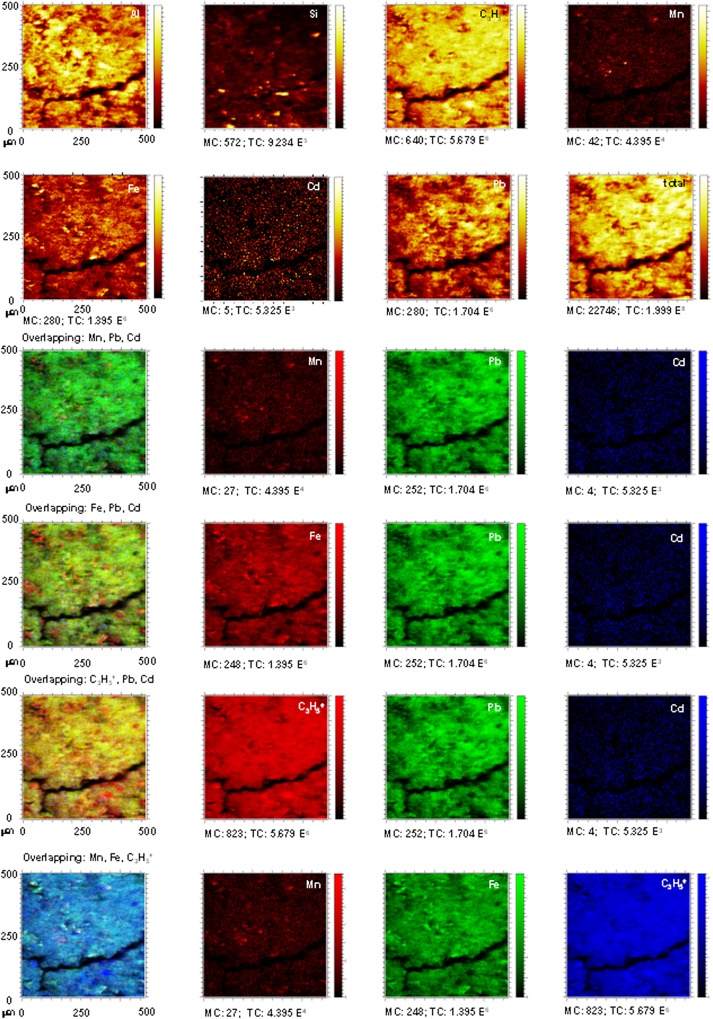
DU.Bw treated with Pb^2+^ and Cd^2+^. TOF-SIMS images of Al, C_3_H_5_^+^, Si, Fe and Mn ions, and overlapping all the signals with Pb^2+^ and Cd^2+^. Images of overlapping: Mn + Pb + Cd; Fe + Pb + Cd; C_3_H_5_^+^ + Pb + Cd; and Mn + Fe + C_3_H_5_^+^ showing the concordance between signals.

The distribution maps corresponding to the untreated DU.Bw horizon show (Fig 3) a very small number of yellow signals, corresponding to Pb^2+^ and Cd^2+^, indicating trace concentrations of both metals. After the treatments, there was an increase in the intensity of the Pb ion signals, resulting in very strong yellow areas that revealed the presence of the fixed Pb^2+^ (Fig 4). However, the intensity of the Cd ion signals remained virtually constant, indicating a scarce retention of the Cd^2+^ in this horizon.

These results coincided with those obtained in the calculation of the competitive retention coefficients for this horizon (Pb^2+^: 0.7053 and Cd^2+^: 0.0392) (Table 2). Therefore a large amount of Pb^2+^ remained fixed, while the added Cd^2+^ mainly remained in the equilibrium solution.

There is a limited association between these Pb^2+^ and Cd^2+^ with the Mn oxides in this soil because there was hardly any overlapping of these two metals with the Mn ions (Figs 3 and 4) before and after treatment.

However, in the case of the overlapping of Fe (red) + Pb (green) + Cd (blue) ions, the results show that there was a very limited association between the Pb^2+^ and Fe ions before the treatment (isolated yellow dots), and that the small amount of Cd^2+^ in the sample overlapped with the Fe ions (magenta areas). After treatment, the fixed Pb^2+^ content was very high, and the distribution map shows (Figs 3 and 4) that the magenta colour has disappeared (there was no overlapping between the Cd ions and the Fe ions) and the appearance of yellow areas. These results show that a high amount of fixed Pb^2+^ overlapped with the signals for Fe and demonstrate that Pb^2+^ was sorbed in Fe-rich components, mainly in the large amounts of oxides contained in this horizon (Table 1 and Figs 3 and 4).

The distribution map corresponding to the overlapping of C_3_H_5_^+^ (red) + Pb (green) + Cd (blue) ions indicates (Fig 3) that in DU.Bw, before the treatment, there was no interaction between the limited signals for Pb^2+^ and those for C_3_H_5_^+^ (organic matter) (Fig 3). However, magenta areas are seen, showing a slight overlap between Cd^2+^ and C_3_H_5_^+^. After the treatment, the distribution map shows an overlap between Pb^2+^ and C_3_H_5_^+^, verifying that Pb^2+^ was strongly linked to the organic matter (yellow areas) (Fig 4). These results indicates a high capacity of organic matter to sorb Pb^2+^ as Lair et al. [39] already have indicated.

Hardly any differences can be seen in the overlaps between Mn (red) + C_3_H_5_^+^ (green) + Fe (blue) (Figs 3 and 4). Cyan areas can be seen in both showing there are mainly interactions between ions of Fe and C_3_H_5_^+^ and to a lesser extent between all three ions (white areas). This fact confirms, together with the previous results, that Fe and Mn oxides and organic matter interacted to decisively influence the sorption of these metals.

**Fig 5:**
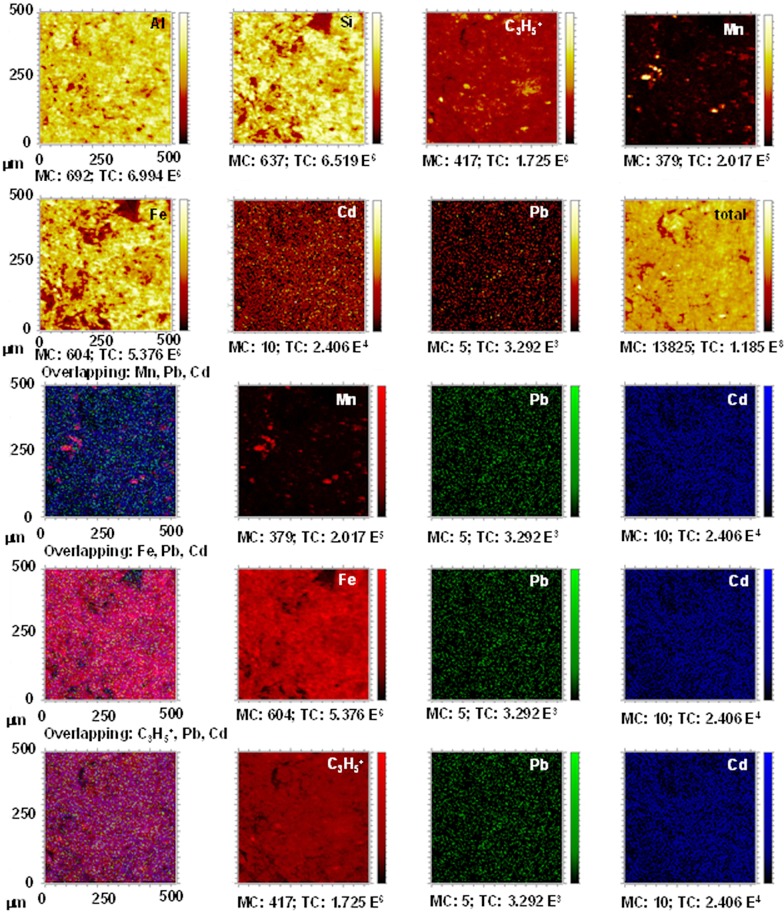
Untreated EL.Bt horizon. TOF-SIMS images of Al, C_3_H_5_^+^, Si, Fe and Mn ions, and overlapping all the signals with Pb^2+^ and Cd^2+^. Images of overlapping: Mn + Pb + Cd; Fe + Pb + Cd; and C_3_H_5_^+^ + Pb + Cd showing the concordance between signals.

**Fig 6:**
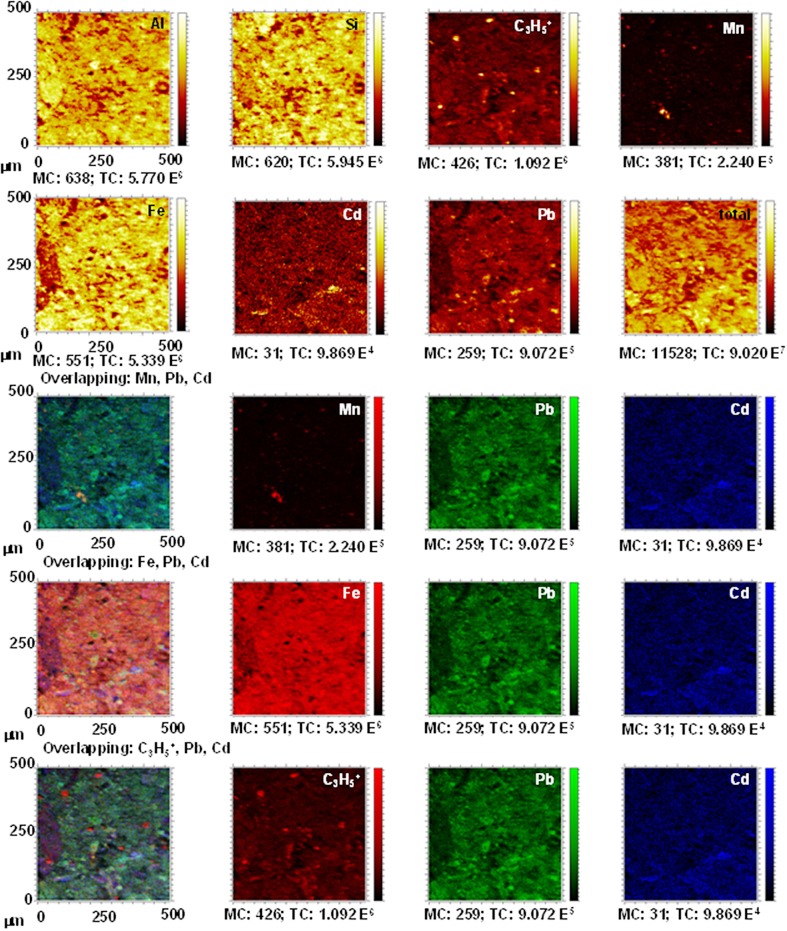
EL.Bt treated with Pb^2+^ and Cd^2+^. TOF-SIMS images of Al, C_3_H_5_^+^, Si, Fe and Mn ions, and overlapping all the signals with Pb^2+^ and Cd^2+^. Images of overlapping: Mn + Pb + Cd; Fe + Pb + Cd; and C_3_H_5_^+^ + Pb + Cd showing the concordance between signals.

The distribution maps for Al, C_3_H_5_^+^, Si, Fe, Mn, and for Pb and Cd ions in the EL.Bw horizon (Figs 5 and 6) also show the coloured distribution maps and the overlapping of the combinations of the ions: Mn + Pb + Cd; Fe + Pb + Cd; and C_3_H_5_^+^ + Pb + Cd.

The distribution maps for the untreated EL.Bw horizon have hardly any yellow signals for Pb^2+^ and Cd^2+^. As a result there was a low content of both metals, much the same as DU.Bw (Fig 5). After the sorption and desorption stages, an increase in the intensity of Pb^2+^ and Cd^2+^ can be seen, giving rise to bright yellow areas. These results are consistent with the results for the competitive retention coefficients of EL.Bt (Pb^2+^: 0.7215, Cd^2+^: 0.2779) and confirm that a large amount of Pb^2+^ and Cd^2+^ remained fixed in the soil, in a higher quantity than in DU.Bw.

The overlapping of the distribution maps for the ions of Mn (red) + Pb (green) + Cd (blue) in soil EL.Bt before treatment show that the small number of green points (Pb^2+^) and blue points (Cd^2+^) do not interact with the red colour (Mn) (Fig 5). After treatment, the signals for Pb^2+^ and Cd^2+^ increased considerably (Fig 6). Some of the signals for the Pb ions (green) overlap with those of Mn (red), resulting in an orange area that shows this interaction. However, the blue signals on the map show that Cd^2+^ did not overlap with Mn (Fig 6).

In this same horizon, there is an overlapping of the signals for Fe (red) + Pb (green) + Cd (blue) ions. This shows that the intensity of the signals for Pb^2+^ (green) and Cd^2+^ (blue), was very low prior to treatment (Fig 5), and increased considerably afterwards (Fig 6). There is also a very weak overlapping between the signals for Pb^2+^ and Cd^2+^ with the signal for Fe.

The distribution map with the overlapping of the signals for C_3_H_5_^+^ (red) + Pb (green) + Cd (blue) ions shows very similar results. No overlapping can be seen for the Pb^2+^ signal with that of the organic matter, and the signal for Cd^2+^ only coincides with that of C_3_H_5_^+^ in a few areas (Figs 5 and 6).

However, the distribution maps for Pb^2+^ and Cd^2+^ after the sorption stages for the EL.Bt horizon show very intense yellow signals in the same areas where the signals for Si were observed. This shows that in this horizon the majority of the Pb^2+^ and Cd^2+^ was fixed on Si-rich minerals, such as vermiculite and chlorite (Table 1). Both minerals have a high CEC and a high capacity to sorb Pb^2+^ [3,13,19].

### Scanning Electron Microscopy

**Fig 7:**
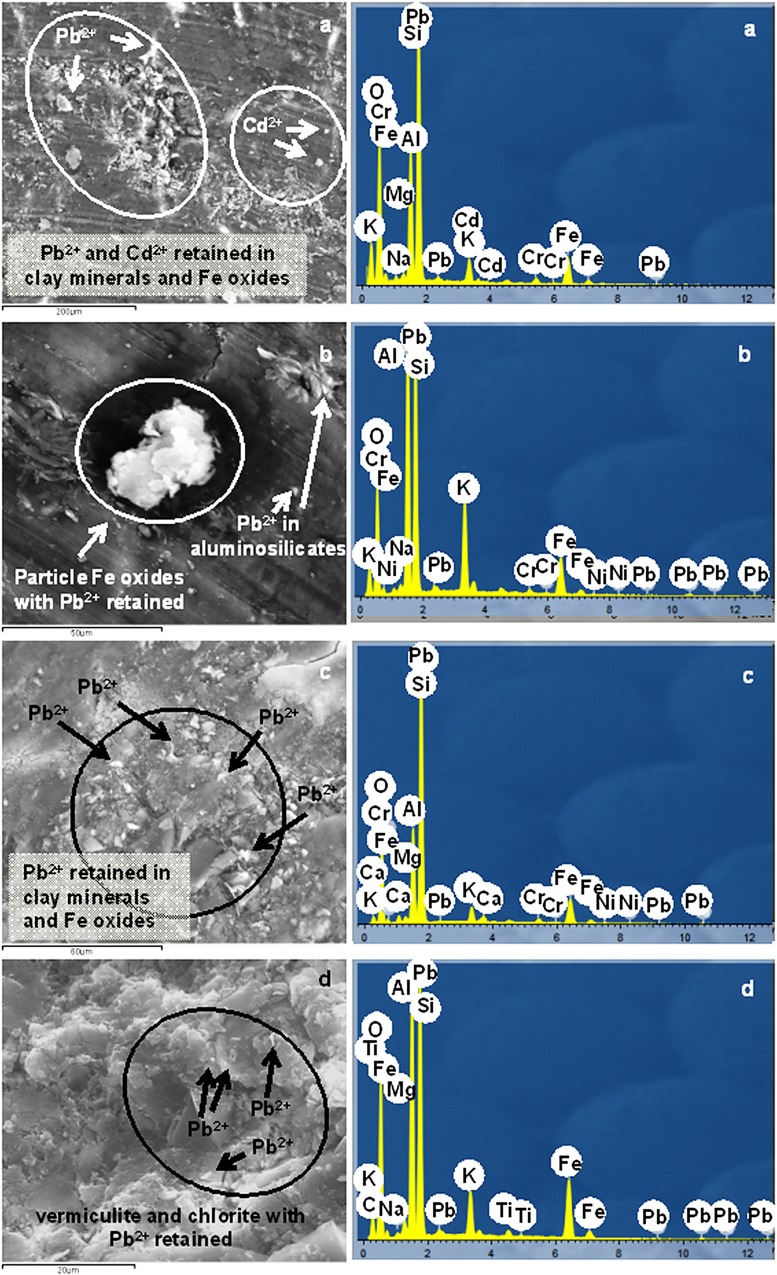
(a): FE-SEM image with Cd^2+^ and Pb^2+^ fixed on Fe amorphous oxides associated with clays (DU.Bw) and typical EDS of clay and Fe oxides containing Cd and Pb; (b) FE-SEM image of Pb^2+^ sorbed on amorphous Fe oxides and aluminium silicates (DU.Bw) and EDS; (c) FE-SEM image and EDS with Cd^2+^ and Pb^2+^ sorbed on clay minerals associated with Fe oxides (MU.Bw): (d) FE-SEM image with Pb^2+^ sorbed on vermiculite, chlorite, and amorphous Fe oxides. Image of vermiculite, chlorite, and Fe oxides (EDS) containing Pb2+.

Fig 7 shows the results of the analyses by Scanning Electron Microscopy with EDS for the soils treated with solutions (3 mmol L^-1^) of Pb^2+^ and/or Cd^2+^, after the desorption stage. The images and spectra are those corresponding to the DU.Bw and MU.Bw horizons, which are representative of all of the others.

The FE-SEM image with its corresponding EDS for soil DU.Bw (Fig 7), after the competitive desorption process, confirmed the results obtained by the procedures described above. This means that Pb^2+^ and Cd^2+^ were mainly fixed in the mineral fraction of the soil and that the Pb^2+^ peaks were much more intense than those of Cd^2+^. This coincides not only with the difference in the Kr values for the retention of this soil, that were 0.705 for Pb^2+^ and 0.039 for Cd^2+^ but also with the high hysteresis of the former (Table 2). Interactions could also be seen between the soil components (Fig 7A), resulting in aggregates of Fe oxides and clays which fixed Pb^2+^. Therefore these results verify the high retention and hysteresis capacities of this ion in this soil. In turn, Cd^2+^ was not detected (Fig 7B) due to it was practically unfixed and returned to the equilibrium solution in the desorption process. All of this confirmed the higher mobility of Cd^2+^, coinciding with the results of Ahmadipour [40] and with the hysteresis indices obtained for this soil (Table 2 and Fig 7B).

The FE-SEM image with its EDS of the MU.Bw horizon after the competitive desorption process (Fig 7C) also shows the fixation capacity of Pb^2+^. However, the peak for Cd^2^ is very small showing its very limited fixation. Pb^2+^ was also found (Fig 7D) fixed on associations of Fe oxides and vermiculite (Table 1), a mineral with a high sorption capacity [33].

It was therefore demonstrated that the association between oxides and clays makes a decisive contribution to the hysteresis and fixing, especially of Pb^2+^, more than the individual components themselves.

The TOF-SIMS and FE-SEM-EDS techniques confirmed the results obtained from the sorption and desorption experiments, and provided more and improved information about the soil components that retain Pb^2+^ and Cd^2+^. They demonstrate that Fe oxides, vermiculite, and chlorite, either separately or by forming associations, have a high affinity for fixing Pb^2+^ and a much lower affinity for Cd^2+^.

## Conclusions

The Kr parameter was validated for comparing the monometallic and competitive sorption and retention capacity and also for evaluating the hysteresis of Pb^2+^ and Cd^2+^ in all of the horizons from five different soils.

Pb^2+^sorption and retention capacities were greater than those of Cd^2+^ in all of the horizons.

The Pb^2+^ sorption hysteresis was higher than that of Cd^2+^.

The pH, ECEC, specific surface area, Fe and Mn oxides, and clay contents—mainly vermiculite and chlorite—were the characteristics that most influenced the sorption and retention of Pb^2+^ and to a lesser extent, of Cd^2+^.

The use of TOF-SIMS combined with FE-SEM / EDS confirmed the results obtained after the sorption and desorption experiments.

Both techniques were effective to verify, firstly, the B horizon components that retained competitive or individually Pb^2+^ and Cd^2+^, secondly, the associations between soil components that interacted with these ions, and lastly, to understand the interactions between these associations, or individual components, and each ion.

## Supporting Information

S1 Fig X ray diffractograms of clay fraction for all horizons. (TIF)

S2 Fig BET isotherms for all horizons (TIF)
