# Supplementary figures and images for: Validation of TOF-SIMS and FE-SEM/EDS Techniques Combined with Sorption and Desorption Experiments to Check Competitive and Individual Pb2+ and Cd2+ Association with Components of B Soil Horizons
Source: PLoS One. 2015 Apr 20;10(4):e0123977. doi: 10.1371/journal.pone.0123977 (PMC4404339; doi:10.1371/journal.pone.0123977)

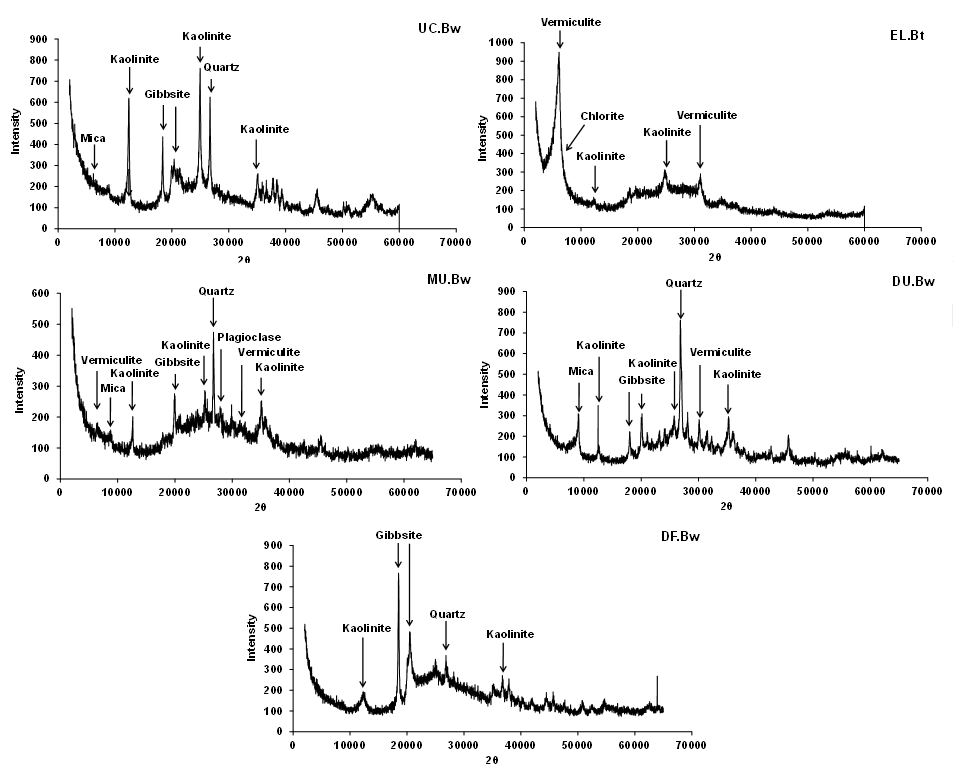

Supplement: S1 Fig — (TIF) [file pone.0123977.s001.tif]

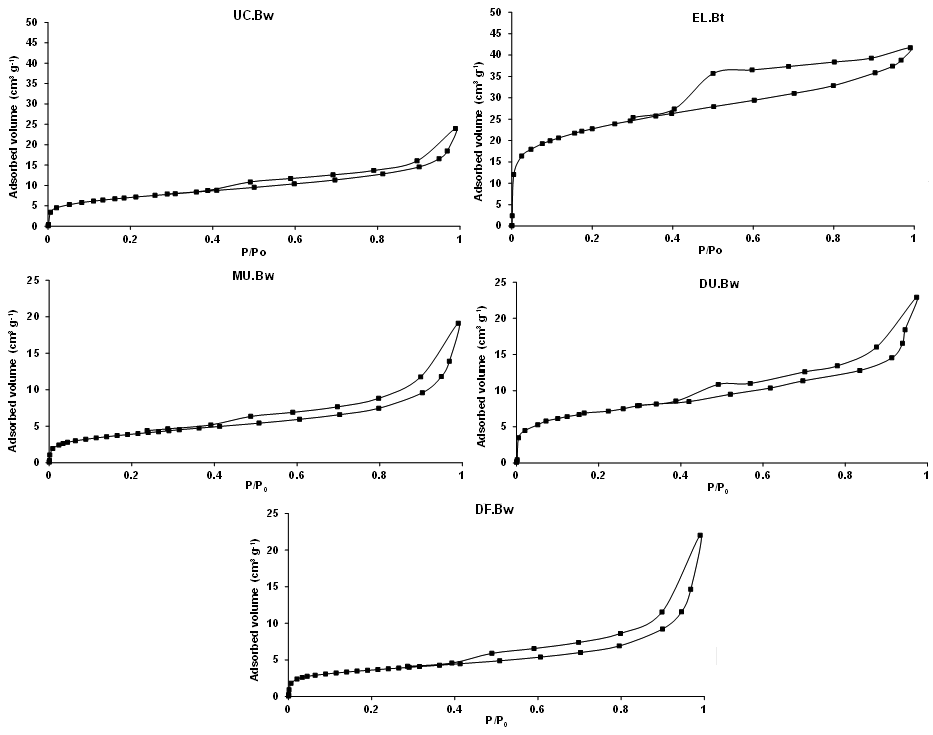

Supplement: S2 Fig — (TIF) [file pone.0123977.s002.tif]
